# Editorial: Rising stars in Food Chemistry

**DOI:** 10.3389/fnut.2022.1019054

**Published:** 2022-09-27

**Authors:** Shuai Chen, Biao Yuan, Fuguo Liu, Yahong Han

**Affiliations:** ^1^School of Public Health, Wuhan University, Wuhan, China; ^2^Department of Food Quality and Safety/National R&D Center for Chinese Herbal Medicine Processing, China Pharmaceutical University, Nanjing, China; ^3^College of Food Science and Engineering, Northwest A&F University, Xianyang, China; ^4^Key Laboratory of Aquaculture Facilities Engineering, Ministry of Agriculture and Rural Affairs, College of Engineering, Huazhong Agricultural University, Wuhan, China

**Keywords:** Food Chemistry, bioactive compounds, fermentation, delivery systems, food nutrition

Recognizing the future leaders of Food Chemistry is fundamental to safeguarding tomorrow's driving force in innovation. This collection showcased the high-quality work of internationally recognized researchers in the early stages of their careers. It includes the preparation of natural products and their health effects, the treatment of food by biological fermentation to enhance its function and activity, the delivery systems for bioactive compounds, food nutrition and toxicology, food safety and detection technology, *etc*.

Natural products have always been an important source of bioactive compounds that were usually used in food field. Polyphenols, polysaccharides, peptides and fatty acids were studied and discussed in this Research Topic. Firstly, scientists studied the extraction of polyphenols and their activity. For example, Xu et al. purified the polyphenol-rich extract from *Pueraria lobata* and investigated systematically its antioxidant activities and its effect on gut microbiota. Results showed that *P. lobata* extract consisted of a large amount of puerarin that exhibited strong antioxidant bioactivity, in addition, it had beneficial and prebiotic effects on the composition and structure of gut microbiota. Qiu et al. analyzed the phenols compositions of waste pomelo seed extract using UPLC-MS/MS and successfully obtained limonin with high concentration in pomelo seed. They illustrated the protect effect of nerve cells by activating the PI3K/Akt pathway and prevented mitochondrial damage from oxidative damage, and effectively reduced Aβ-induced neurotoxicity. Lin et al. used oxidative damaged human umbilical vein endothelial cells (HUVECs) as a model and investigated the regulatory effect of procyanidins from sea-buckthorn (SBP) on HUVECs. It provided a theoretical foundation for further research on natural bioactive compounds to exert antioxidant activity in the body. Secondly, the extraction and function of polysaccharides have also been studied, Wang, XU et al. found that the purified *G. lemaneiformis* polysaccharide (PGP) pretreatment could decrease senescence-associated β-galactosidase (SA-β-gal) positive cells and prevent the formation of senescence-associated heterochromatic foci (SAHF) induced by H_2_O_2_ in a dose-dependent manner. It is speculated that PGP may delay aging by downregulating the expression of p21 and p53 genes. Liu, Zhang et al. screened a homogeneous and high molecular weight polysaccharide (named RGLP-1) with the greatest immune-regulatory activity *in vitro* after double purification with ultrafiltration membrane and Sephacryl S-500 HR and explored the structure-activity relationship of the polysaccharide from *Ganoderma lucidum*. Wang, Cai et al. reviewed effects of natural polysaccharides on inhibiting the cell proliferation and growth, regulating the tumor cell cycle, inducing apoptosis, suppressing the tumor cell migration and invasion, improving the immunomodulatory activities, and enhancing the efficacy of chemotherapy (cisplatin) in ovarian cancer. In recent years, peptides have attracted more and more attention from researchers. Jia et al. demonstrated that pea peptide combined with resistance exercise training markedly promoted skeletal muscle growth. Furthermore, they found that the oligopeptide with amino acid sequence of LDLPVL presented a more significant proliferation of C2C12 cells than other oligopeptides. Zu et al. explored the physicochemical properties and biological functions of silver carp scale peptide (SCSP), its molecular-weight fractions SCSP-I, II, and III obtained by nanofiltration were assessed for their solubility, emulsibility, free radical scavenging ability, effect on the proliferation of mouse B16 cells. This work provided a data base for the development of SCSP and increases the possibility of its application. Wang, Qiao et al. summarized the research progress of milk fat globule membrane (MFGM), including separation, identification, and functional properties. MFGM can be separated and prepared from fresh milk, cream, casein and cream whey. The composition of MFGM is complex, mainly composed of polar lipids and membrane-specific proteins. MFGM has good Emulsifying, foaming, and water holding properties, as well as various biological activities such as immunomodulatory activity, anti-enteritis activity, anti-tumor activity, and promoting cell growth and differentiation. This paper can provide some guidance for the development and utilization of MFGM. Fatty acids play an important role in metabolism and gut microbiota, Xiao et al. found that DHA significantly reduced the deposition of amyloid β-peptide in the brain and inhibited the production of nerve fibers, thereby increasing cognitive abilities in the mice with Alzheimer's disease. Zhang, Su et al. demonstrated that a 14-day Laminaria japonica supplementation, one of the most widely consumed commercial edible seaweeds, could balance firmicutes/bacteroidetes ratio and strengthen the utilization of alginate so to enhance the production of short chain fatty acid. Some related studies of other natural bioactive products are also included in this collection. Jiang et al. found that ginseng saponin Rb1 can effectively restore the depressive-like behavior in chronic social defeat stress (CSDS) induced model mice, mediated in part by the normalization of oxidative stress levels. The suppression of neuroinflammation is mediated by the regulation of SIRT1-NLRP3/Nrf2 pathways. Rb1 is a novel therapeutic candidate for treating depression.

Fermentation can also be used to produce bioactive compounds. Yang, Gao et al. used three lactic acid bacteria strains to ferment *Porphyra yezoensis* sauces, including *Lactobacillus fermentum, Lactobacillus casei, Streptococcus thermophilus*, and the mixed strains, analyzed the fermentation characteristics, antioxidant capacity *in vitro*, sensory properties, and flavoring substances of fermented *P. yezoensis* sauces. They found that the fermented *P. yezoensis* sauce possessed greater DPPH radical scavenging activity and ferric-reducing ability power than the unfermented P. yezoensis. In addition, the whitening, moisturizing, anti-aging activities, and skincare evaluation of selenium-enriched mung bean fermentation broth was investigated by Wei et al., and a clinical trial was conducted on 31 Chinese women aged 25–60 years, the test of the Se-MBFB mask showed that after 4 weeks of using the Se-MBFB facemask, the faces of the participants became whiter with reduced wrinkles and increased moisture content. Yin et al. identified two potential allergens, glutaredoxin and oleosin-B2, in Brassica napus bee pollen using mass spectrometry-based proteomics analyses, and used bioinformatics to predict their antigenic epitopes. Fermentation could potentially alleviate allergenicity, while also positively affecting nutritional properties of B. napus bee pollen.

Bioactive compounds have various health effects, however, their physicochemical properties are unstable and their bioavailability is low. Developing and fabricating the delivery systems can solve these problems. Liu and peng has prepared a high-performance hyaluronic acid-black rice anthocyanins (HAA) nanocomposite particles by a simple crosslinking method. HAA nanocomposite particles can effectively improve black rice anthocyanins' stability and activity, creating an ideal new material for inhibiting xanthine oxidase activity. Qiao et al. has prepared phycocyanin-sodium alginate/lysozyme complex (PC-SLC), the complex formed by the mass ratio of SA-LZM of 0.1 showed the highest PC encapsulation rate (89.9 ± 0.374%). Compared with free phycocyanin (PC), its thermal stability was obviously improved, and could exist stably in simulated gastric fluid for 2 h and be slowly digested in simulated intestinal fluid, which helped to promote the absorption of nutrients in the intestinal tract. Wang, Wang et al. analyze the stability and interaction mechanism of the complex glycosylated soy protein isolate prepared using soy protein isolate, inulin-type fructans and lutein. They showed that glycosylation reduced the fluorescence intensity and surface hydrophobicity of soy protein isolate but improved the emulsification process and solubility. Liu, Yang et al. developed curcumin-encapsulated zein/polysaccharide complex nanoparticles as a therapeutic agent against colorectal cancer (CRC), including gum Arabic-zein-curcumin (GA-Zein-CUR), hyaluronic acid- zein-curcumin (HA-Zein-CUR), and pectin-zein-curcumin (PC-Zein-CUR). Results showed that HA-Zein-CUR with the highest encapsulation efficiency and loading capacity, inhibited cell viability and colony formation, exhibited higher cellular uptake of CUR, enhanced pharmacokinetic properties of intragastric administration, delivery and accumulation of curcumin in major organs/tissues, in particular CRC tumors and colon. Zhang, Hao et al. studied the impact of carboxymethyl cellulose (CMC) on the physicochemical stability, rheological property, and *in-vitro* digestion of soybean protein isolate (SPI)-stabilized rice bran oil (RBO) emulsions. RBO emulsions stabilized by SPI and various contents of CMC were prepared, it showed that the chemical stability, free fatty acids release rate and bioavailability of the emulsion added with CMC was improved compared with the SPI-stabilized RBO emulsion. Yang, Wei et al. developed an antioxidative trimetallic complex with high stability by interacting Ca^2+^, Fe^2+^, and Zn^2+^ with the biodegradable ligands from Maillard reaction and then investigated the effect of the complex on the mineral contents of apple. The results showed that mineral nutrients of Ca, Fe, and Zn in apple increased significantly through surface spraying of the metal–integrated complex, and the efficiency is much higher than the traditional inorganic salts and single chelated metals.

In addition to bioactive compounds, the proportion of carbohydrates and lipids in the diet also has a regulatory effect on human health. Mei et al. reported that the mediterranean diet / low-carbohydrate (MED/LC) diet model is a good treatment for overweight polycystic ovary syndrome (PCOS) patients, significantly restoring their menstrual cycle, improving their anthropometric parameters and correcting their disturbed endocrine levels, and its overall effectiveness is significantly better than the low-fat diet model systematically Chen et al. characterized the diversity of fungal communities, chemical composition, antioxidant activity, and taste quality of Fu brick tea samples from five different regions of China, and assessed the relationship between bioactive metabolites and fungal communities and the key chemical substances contributing to the variations in antioxidant activity and taste profile.

Food safety is also an important factor affecting human health. Zhao et al. reviewed the exposure to occupational, environmental, and daily acrylamide (ACR) contamination in food. Moreover, acrylamide metabolism and the potential mechanism of acrylamide-induced neurotoxicity were discussed, with particular focus on the axonal degeneration of the nervous system, nerve cell apoptosis, oxidative stress, inflammatory response, and gut-brain axis homeostasis. Perchlorate, commonly available in drinking water and food, acts on the iodine uptake by the thyroid affecting lipid metabolism. High-fat diets leading to various health problems continually raise public concern. In the study of Wang, Song et al., liver lipid metabolism profiles and metabolic pathways were investigated in C57BL/6J mice chronically exposed to perchlorate using targeted metabolomics. Perchlorate low, medium and high dose groups were identified with 11, 7 and 8 significantly altered lipid metabolites compared to the control group, respectively. The improvement of detection technology is an important guarantee to maintain food safety. Fan et al. developed and established a UPLC-MS/MS method for the detection of tropomyosin in shrimp and crab. While Guo et al. successfully developed a method for the identification and quantification of 103 common veterinary drug residues in milk and dairy products.

Overall, the collection highlighted research by leading scientists of the future across the entire breadth of Food Chemistry, and present advances in theory, experiment and methodology with applications to compelling problems.

## Author contributions

SC, BY, FL, and YH prepared, checked and revised the manuscript, and approved the submitted version. All authors contributed to the article and approved the submittedversion.

## Funding

This study was supported by the Fundamental Research Funds for the Central Universities (2042021kf0042); the Beijing Engineering and Technology Research Center of Food Additives, Beijing Technology and Business University (BTBU); the Postdoctoral Innovative Research Position of Hubei Province; China Postdoctoral Science Foundation (2022M712473 and 2019M662659); Hubei Provincial Natural Science Foundation of China (2020CFB314); the National Natural Science Foundation of China (No. 31901699).

## Conflict of interest

The authors declare that the research was conducted in the absence of any commercial or financial relationships that could be construed as a potential conflict of interest.

## Publisher's note

All claims expressed in this article are solely those of the authors and do not necessarily represent those of their affiliated organizations, or those of the publisher, the editors and the reviewers. Any product that may be evaluated in this article, or claim that may be made by its manufacturer, is not guaranteed or endorsed by the publisher.

